# Retraction Note: Determining the role of innovative teaching practices, sustainable learning, and the adoption of e-learning tools in leveraging academic motivation for students’ mental well-being

**DOI:** 10.1186/s40359-025-02871-1

**Published:** 2025-05-22

**Authors:** Jiuxiang Li, Rufeng Wang

**Affiliations:** 1 Social Studies Education, Sunchon National University, Sunchon, 57922 Jeollanam-do Korea; 2 College of Physical Education, South-Central Minzu University, Wuhan, 430074 Hubei China

**Retraction Note: BMC Psychology (2024) 12:163**

10.1186/s40359-024-01639-3

The editor and the publisher have retracted this article. The article was submitted to be part of a guest-edited issue. An investigation by the publisher found a number of articles, including this one, with a number of concerns, including but not limited to compromised editorial handling and peer review process, inappropriate or irrelevant references or not being in scope of the journal or guest-edited issue. Based on the investigation’s findings the editor therefore no longer has confidence in the results and conclusions of this article.

The authors have not responded to correspondence regarding this retraction.

